# Comparison of Effectiveness regarding a Culinary Medicine Elective for Medical Students in Germany Delivered Virtually versus In-Person

**DOI:** 10.3390/nu15194281

**Published:** 2023-10-08

**Authors:** Selina Böttcher, Louisa Josefa Schonebeck, Laura Drösch, Anna Manuela Plogmann, Can Gero Leineweber, Seraphina Puderbach, Charlotte Buhre, Christoph Schmöcker, Uwe Neumann, Thomas Ellrott

**Affiliations:** 1 Medical Department, Division of Gastroenterology, Oncology, Hematology, Rheumatology and Diabetes, ukrb, Brandenburg Medical School, Theodor Fontane, 16816 Neuruppin, Germany; can.leineweber@mhb-fontane.de (C.G.L.); seraphina.puderbach@mhb-fontane.de (S.P.); charlotte.buhre@mhb-fontane.de (C.B.); christoph.schmoecker@mhb-fontane.de (C.S.); 2 Institute for Nutrition and Psychology, Georg-August-University Göttingen Medical Centre, Humboldtallee 32, 37073 Göttingen, Germany; l.schonebeck@stud.uni-goettingen.de; 3 Department of Internal Medicine I, Gastroenterology, Hepatology, Endocrinology, Rheumatology, and Infectious Diseases, University Hospital Regensburg, 93053 Regensburg, Germany; laura.droesch@klinik.uni-regensburg.de; 4 Medical Centre, Justus-Liebig-University Gießen, 35390 Gießen, Germany; anna.m.plogmann@med.uni-giessen.de; 5 Faculty of Health Sciences, Joint Faculty of the Brandenburg, University of Technology, Brandenburg Medical School and University of Potsdam, 14469 Potsdam, Germany; 6 Culinary Medicine Germany e.V., 48341 Altenberge, Germany; uwe.neumann@culinarymedicine.de

**Keywords:** culinary medicine, teaching kitchen, nutrition education for health professionals, hands-on cooking instructions, interprofessional education, nutrition knowledge, behaviour change, diet-related diseases, counselling competencies, patient care

## Abstract

(1) Background: The Culinary Medicine elective at the German medical schools of the universities of Göttingen, Giessen, and Brandenburg is a teaching kitchen-based elective aimed at training medical students on how to improve patient counselling on nutrition and lifestyle medicine topics. This curriculum was either delivered virtually (2021) or in-person (2022/2023). Changes in teaching effectiveness were evaluated. (2) Methods: The elective included seven modules in the teaching kitchen for 3 h each. It consisted of a short introduction and a hands-on interactive cooking part illustrating important dietary principles in different disease groups. The elective was conducted virtually in 2021 in a fully interactive setup using videoconference tools. Students in this cohort attended from their private kitchens whereas students in the in-person cohort (2022/2023) attended the same classes in the teaching kitchen. Standardized comparative self-assessment questionnaires on counselling competencies, nutrition knowledge, eating habits, and mental well-being (WHO-5) before and after the elective were used to determine teaching effectiveness. Paired and unpaired *t*-tests were performed to evaluate results. (3) Results: A total of 70 students (mean semester 6.3) were included in the virtual cohort, and 80 students (mean semester 6.3) were in the in-person cohort. In both, counselling competencies on 25 nutrition and lifestyle medicine topics increased significantly. Significant changes also occurred in most nutrition knowledge categories. Subjective well-being as well as personal attitudes towards nutrition counselling in medical practice improved significantly during the elective. Healthy eating habits improved in both groups as students ate significantly less unfavourable foods. There were no significant differences between the two groups apart from minor differences in nutrition knowledge. (4) Conclusions: The elective in Culinary Medicine improved students counselling competencies, nutrition knowledge, attitudes, well-being, and eating habits with no relevant difference between virtual and in-person teaching.

## 1. Introduction

Diet-related diseases are one of the greatest challenges for healthcare systems worldwide. Direct costs for a diagnosis and therapy but also indirect costs due to sick leave and early retirement especially in industrialized societies are immense. In addition, there are numerous handicaps and suffering for those affected including reduced participation in social life. In 2015 alone, 4 million deaths and 120 million disability-adjusted life years were caused worldwide by excess body weight and consecutive diseases [1]. About one in five deaths globally can be linked to insufficient diet quality [2]. Cardiovascular disease and metabolic disorders promoted with poor dietary patterns are the main driver of this association followed by cancer.

Despite these drastic consequences of diet-related diseases, nutrition and lifestyle medicine has barely found its way into medical schools [3,4]. Lifestyle medicine is a new discipline of healthcare that focuses on promoting and implanting healthy behaviours and lifestyle changes to prevent, manage, and treat chronic diseases and improve overall well-being [5,6]. On average, European medical students receive 14 h of nutritional instruction throughout their studies [7]. Even though physicians are often the first point of contact for dietary and lifestyle changes, they lack knowledge and counselling competencies to make well-founded recommendations that also fit into patients’ lifestyles and routines. This can be explained with the lack of nutrition-related education and training during their studies, a problem that can be solved [8,9,10]. The global impact of diet-related disease demands innovative nutrition education for physicians as well as other health professionals. Also, an improved interprofessional collaboration across disciplines, especially with registered dietitian nutritionists but also with nurses, is necessary [11].

There are several approaches to overcome these education and training gaps. Culinary medicine (CM) has emerged among the most promising: “Culinary medicine is a discipline and training modality within clinical and public health education that provides medical trainees (e.g., medical students, nursing students, dietetic interns), healthcare professionals and community members with experiential, food-based nutrition knowledge and the culinary skills needed for implementation (Razavi AC et al., 2023) [12]”. CM takes place in teaching kitchens to transfer theoretical knowledge to practical skills that can be implemented in personal and professional everyday life [13].

The term “teaching kitchen” stands not only for the location itself but also for the hands-on teaching method with the addition of practical cooking skills and nutrition education, and with special attention to food science. The format teaching kitchen has a special focus on embedding recommendations into a healthy lifestyle with physical activity, psychosocial aspects to healthy eating behaviours, as well as strategies to support lifestyle changes (e.g., motivational interviewing) [14]. Therefore, teaching kitchens can provide an excellent opportunity to teach nutrition and lifestyle medicine to professionals as well as the community [15].

While teaching CM for medical students is already established in the United States [16,17], the first locally adapted CM elective in Germany was developed by the Institute for Nutrition and Psychology at the Georg-August-University Göttingen, University Medical Centre, in cooperation with Culinary Medicine Germany e.V. (formerly CookUOS e.V.), based on methodological preparatory work by Neumann (2018) [18]. It was established in 2020 as an elective for medical students at the University of Göttingen, University Medical Centre [19]. Since 2021, it is also used at the university medical centres of Gießen and Brandenburg.

The benefits of hands-on CM classes for medical students regarding counselling competencies, nutrition knowledge, and improved dietary habits have already been proven [20,21].

Due to the COVID-19 pandemic, CM classes had to be switched to virtual platforms like Zoom/Teams/WebEx, where the professional teaching staff hosts the virtual CM classes from a teaching kitchen. The students use their home kitchens. Teaching success using virtual platforms has been shown recently [12].

The aim of this study was to compare the effectiveness between the same CM elective for medical students in Germany delivered virtually versus in-person using standardized comparative self-assessment questionnaires on counselling competencies, nutrition knowledge, eating habits, personal attitudes towards nutrition counselling in medical practice, and mental well-being (WHO-5) before and after the elective.

## 2. Materials and Methods

### 2.1. Design

The study was carried out at the university medical centres of the University of Göttingen (UMG), the Brandenburg Medical School, Theodor Fontane (MHB), and the University of Gießen, Justus Liebig (JLU).

There were two study populations: a virtual cohort during the COVID-19 pandemic and an in-person cohort after pandemic measures were lifted. The cohorts were not randomized as all teaching during the COVID-19 pandemic had to be virtual. Since the end of the pandemic and from April 2022 onward, in-person teaching of the CM curricula has resumed at all three locations.

### 2.2. Participants

Participants were medical students enrolled in any semester for human medicine at one of the three participating universities. Inclusion criteria were participation in the Culinary Medicine electives. Exclusion criteria were non-applicable.

### 2.3. Culinary Medicine Elective

The newly developed German CM curriculum was offered as an elective and included 28 lecture hours (45 min. each). It consisted of seven modules and was held once a week (4 lecture hours) for 7 weeks or as a block of 4 to 5 consecutive days (7 lecture hours per day). Each module included an interactive lecture, discussion of the recipes, supervised hands-on cooking, tasting, and a shared meal with discussion. About five to seven recipes were cooked per module. These showed the main dietary principles in the different disease groups (see below). Printed recipe cards included an illustrated easy-to-follow step-by-step manual.

The CM curriculum was based on the German consensus paper “manual of nutritional therapy in patient care” (LEKuP) [22]. The seven modules cover the following topics: healthy diet, malnutrition, dietary therapy of cardiovascular disease and metabolic diseases part I and II, dietary management of gastrointestinal diseases, dietary therapy of kidney diseases, and dietary therapy for inflammatory rheumatic, orthopaedic, neurological, and pulmonal diseases. The elective was conducted by an interprofessional team including registered dietitian nutritionists, chefs specially trained in nutrition and dietetics, and tutors who were medical students and who had already taken part in the CM curriculum and had been additionally trained. A physician with experience in CM ideally completed the interprofessional team.

As the elective was conducted virtually in 2021 using a Zoom/Teams/WebEx platform in a fully interactive setup with cameras and audio, the associated recipes were distributed before the classes. In this cohort, students themselves had to shop for groceries and prepare their private kitchens for cooking and for the videoconference. The in-person cohort (2022/2023) attended the CM classes in the teaching kitchen where all of the five to seven recipes were cooked. Virtual participants only cooked one or two recipes in their own kitchens but watched the preparation of all other recipes in the videoconference. The amount of time spent on cooking was approximately 2 h in both formats. The difficulty level of the recipes varied depending on the recipe, but in general, their levels were easy to medium. Group size in both formats was between 8 and 14 students. Although virtual classes could theoretically be larger, it was easier to fully interact with all participants, when everyone could be seen clearly on the main videoconference screen with sound and vision. Therefore, class size in the virtual format was the same as in the in-person format.

Student total time requirements differed depending on the teaching format. Table 1 shows the calculation.

The CM curriculum was offered as an elective at the above-mentioned three universities and was therefore considered a passive explanatory variable. It was not specifically allocated to participants.

### 2.4. Evaluation Tools

To determine teaching effectiveness in both groups before and after the elective course, a standardized comparative self-assessment questionnaire on counselling competencies, personal attitudes towards nutrition counselling in medical practice, nutrition knowledge, mental well-being (WHO-5), and eating habits was developed as a web-based LimeSurvey.

The questions about counselling competencies and personal attitudes towards nutrition counselling in medical practice were derived from Razavi et al. [20]. Twenty-five different consulting topics involving different diets as well as nutrition principles for various diseases were included. As the DASH-Diet is not well known in Germany, dietary recommendations for patients with arterial hypertension according to the LEKuP [22] were used instead.

Sixteen multiple choice questions about the dietary therapy of different diet-related diseases according to the LEKuP were used to assess the students’ nutrition knowledge.

The WHO-5 well-being index was used to measure mental well-being.

Eating habits were measured using a five-point Likert scale, which asked about the frequency of consumption per week in 12 various food groups. This was based on the 10 guidelines for healthy eating published by the German Association for Nutrition/DGE [23].

Participation in the evaluation was voluntary. The pre-survey was completed within 2 weeks before the students started the course. The post-survey was completed within 2 weeks after the students finished the course.

### 2.5. Data Collection and Analysis

Data from students at UMG, MHB, and JLU were collected using LimeSurvey. LimeSurvey is a free and open source online statistical survey web app written in PHP based on different databases. It is distributed under the GNU General Public License. Participants created an individual pseudonym at the beginning of the LimeSurvey that was used in the pre- and post-survey to identify corresponding data sets/pairs. Afterwards, data were transferred to Microsoft Excel and IBM SPSS Statistics.

Data collection and statistical analyses were made using LimeSurvey (Version 3.24.2 + 201020), Microsoft Excel Software systems (Version 16.75.2), and IBM SPSS Statistics (28.0.1.0). Statistical calculation, *t*-tests, and graphs were created with IBM SPSS Statistics (28.0.1.0) and GraphPad Prism 10 10.0.2 (171).

Normal distribution of data was assumed using quantile–quantile plots.

Paired *t*-tests were used to compare pre- and post-survey results in both the virtual and in-person cohorts. The comparison between in-person and virtual was performed with unpaired *t*-tests referring to the questionnaire sections on counselling competencies, eating habits, and the WHO 5 well-being index. Statistical significance was assumed with *p*-values < 0.05.

Effect size was determined with Cohen’s d. The following interpretations were assumed: there was no effect at d < 0.2, a small effect at d = 0.2–0.49, a medium effect at d = 0.5–0.79, and a large effect at d ≥ 0.8 [24].

For the data analysis, the statements were interval-scaled into a Likert scale.

The virtual cohort consisted of 70 medical students. The data were collected between February 2021 and September 2021. The in-person cohort consisted of 80 medical students. These data were collected between April 2022 and July 2023.

### 2.6. Ethics Approval

The study was approved by the ethics committee of the UMG, University Medical Centre, on 23 September 2022 under the application number 25/9/22.

## 3. Results

### 3.1. Participants

The initial virtual cohort consisted of 91 medical students completing the CM curriculum. Complete data sets of the voluntary pre- and post-surveys were obtained from 70 medical students (76.9%). These were used for the statistical analysis. The initial in-person cohort consisted of 149 medical students completing the CM curriculum. Of these, we obtained 80 completed voluntary pre- and post-surveys for the statistical analysis (53.6%). Exact details of the student characteristics can be found in Table 2.

All participants were medical students and chose CM as one of two mandatory electives for their medical degree. Most students in both cohorts were clinical students with only a few pre-clinical students. Un-paired *t*-tests were performed to test for statistical differences between the two cohorts (Table 2).

### 3.2. Counselling Competencies

CM classes had significant positive effects on counselling competencies in all 25 categories (Table 3) and in both cohorts (virtual: *p* ≤ 0.001; in-person: *p* < 0.001). Even if the in-person cohort sometimes achieved greater changes in mean values (virtual: 0.51–1.46; in-person: 0.7–1.7), the unpaired *t*-tests showed that there was no significant difference between cohorts (Table S2—Supplementary Materials).

**Figure 1:**
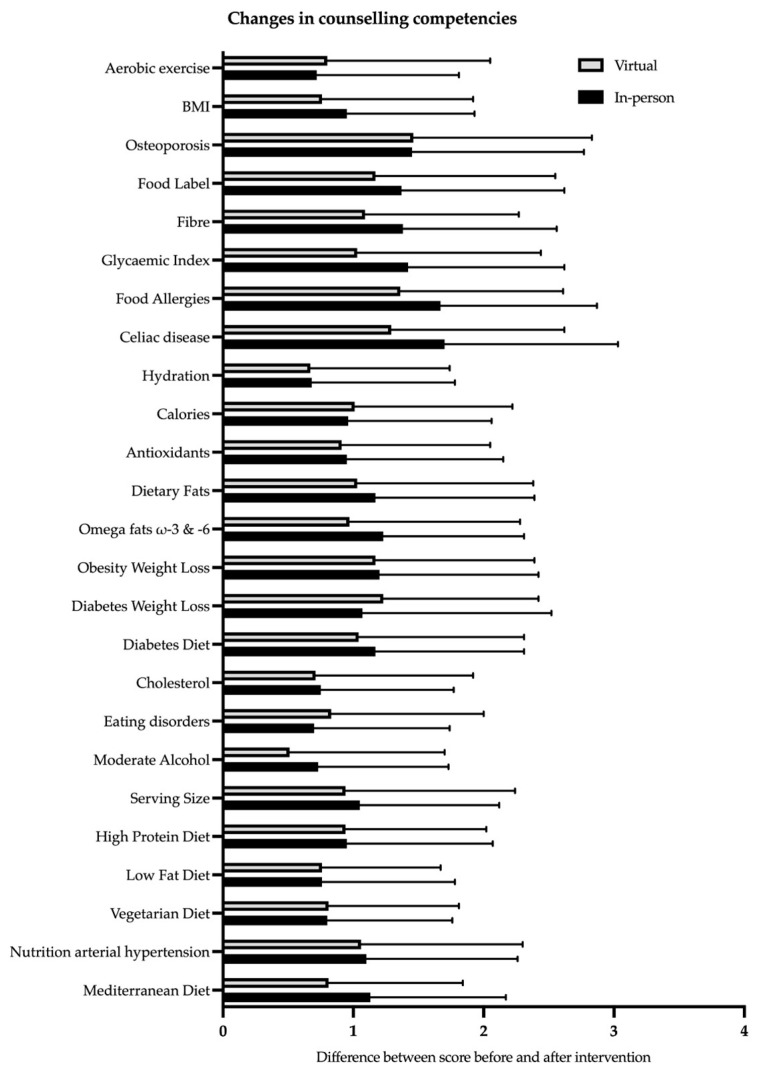
Improvements in counselling competencies on a scale from 0 to 5.

Figure 1 shows the clear improvements that are independent of the CM teaching format. The mean increase in counselling competencies across all categories was 31% in the virtual cohort and 34% in the in-person cohort.

### 3.3. Attitudes towards Nutrition Counselling in Medical Practice

There was a significant positive shift in personal attitude towards nutrition counselling in medical practice in all statements and in both cohorts (Table 4): “Physicians can influence their patients’ eating habits if they take the time to discuss the problem with patients” (virtual *p* = 0.007; in-person *p* = 0.036), “counselling on nutritional issues should be part of every medical consultation, just like therapy and diagnosis” (virtual *p* = 0.019; in-person *p* = 0.006), and “specific recommendations for changing eating behaviours can help patients improve their eating habits” (virtual *p* = 0.001; in-person *p* ≤ 0.001). The mean difference in the in-person cohort was larger (virtual = 0.1–0.33; in-person = 0.22–0.36), but the difference between both cohorts was not significant (Table S4—Supplementary Materials).

### 3.4. Nutrition Knowledge

The pre-survey students in the virtual cohort increased their nutrition knowledge by 48% compared to 41% in the in-person cohort (Table 5). There was a significant difference between the virtual and in-person cohort in five questions (see Table S6 in Supplementary Materials). The virtual cohort scored significantly better in three questions about carbohydrate percentage (*p* = 0.002), cereals for celiac disease (*p* = 0.008), and fructose malabsorption (*p* = 0.012). The in-person cohort scored significantly better in two questions about calcium oxalate stones (*p* ≤ 0.001) and omega-3 fatty acids (*p* ≤ 0.001).

### 3.5. WHO-5 Well-Being Index

There was significant improvement in well-being in both the virtual (mean score before virtual at 15.2 to mean score after virtual at 16.6) and in-person cohort (mean score before in-person at 14.7 to mean score after in-person at 16.2) (virtual *p* ≤ 0.001; in-person *p* = 0.004), but no significant difference between both cohorts (Table S9 in Supplementary Materials).

### 3.6. Eating Habits

**Figure 2:**
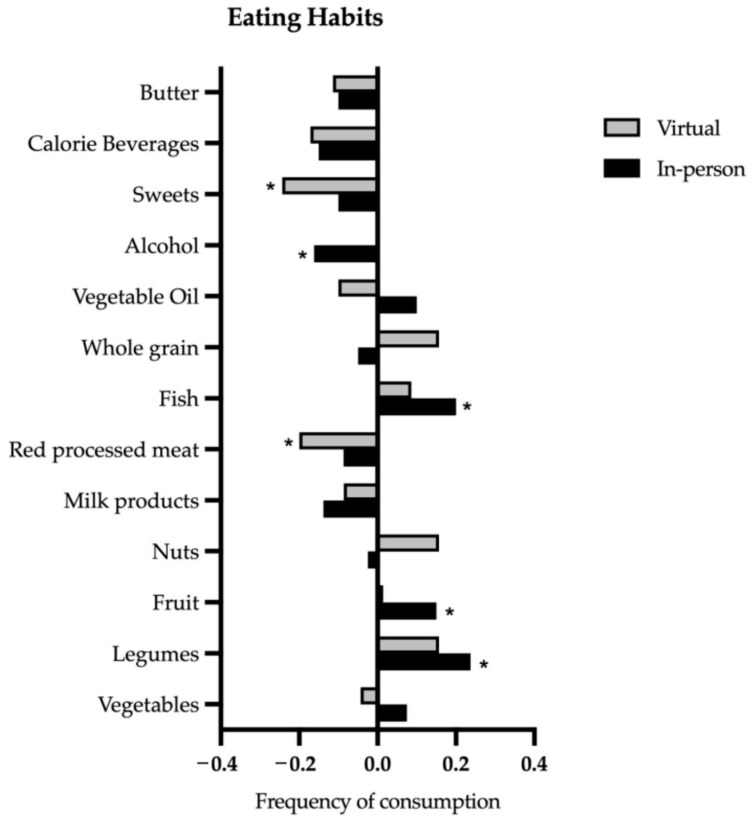
Differences in consumption frequency (* *p* ≤ 0.012 compared to baseline).

The consumption frequency per week in 12 different food groups was assessed using a five-point frequency scale before and after the CM curriculum (Figure 2).

In the virtual cohort, a significant decrease in the consumption of red processed meat (*p* = 0.012) and sweets (*p* = 0.006) was seen. In the in-person cohort, there was a significant increase in legumes consumption (*p* = 0.007) and non-fried fish (*p* ≤ 0.001), as well as a significant decrease in alcohol consumption (*p* = 0.001). There was no significant difference between both cohorts in the single food categories except in alcohol consumption (*p* = 0.026).

We divided the 12 food groups into favourable (vegetables, legumes, no fried fish, vegetable oils, fruit, whole grain foods, and nuts) and unfavourable foods (red processed meat, caloric beverages, alcohol, sweets, and butter).

The virtual cohort ate significantly less favourable foods (*p* ≤ 0.001), while the in-person cohort ate more. However, they did not show a significant difference (*p* = 0.07). Both groups ate significantly less unfavourable foods (virtual *p* ≤ 0.001; in-person *p* = 0.003). There was a significant difference between the in-person and virtual cohort regarding the consumption of favourable foods before (*p* = <0.001) and after (*p* = 0.024) the CM classes. Before CM intervention, the virtual cohort consumed significantly more favourable foods than the in-person cohort; after CM intervention, the in-person cohort consumed significantly more favourable foods than the virtual cohort (Table 6). There is no significant difference between cohorts regarding the consumption of unfavourable foods.

There was no significant difference in both cohorts in home cooking frequency after participating in the CM curriculum, which itself included additional cooking.

## 4. Discussion

Our study design is consistent with other evaluations of CM curricula for medical students: Razavi et al. (2020), Jaroudi et al. (2018), Pang et al. (2019), Rothman et al. (2020), and Wattick et al. (2022). In all studies, a pre-/post-survey with different scales was used to measure teaching success [20,25,26,27,28].

The elective in CM improved students counselling competencies, nutrition knowledge, attitudes towards nutrition counselling in medical practice, mental well-being, and eating habits with no relevant difference between virtual and in-person teaching. Counselling competencies and nutrition knowledge are most important for patient care. Especially counselling competencies improve interpersonal and communication skills in lifestyle medicine. Physicians can communicate more effectively with patients, their families, and other health professionals in the broad context of diet-related diseases.

Newman et al. (2023) recently described how curricula in CM improve patient counselling skills:

“Culinary medicine programs give medical students the experience necessary to translate nutrition knowledge learned in typical medical school curricula into practical advice. Culinary experiences allow medical students to provide their patients with individualized food and nutrition ideas that are easier to translate into actionable changes than the typical generalities of healthy eating imparted during many patient visits [21]”.

Systems-based practice skills are also improved because medical students learn to use interprofessional collaboration across disciplines in the CM classes, especially with registered dietitian nutritionists, to provide the best patient care possible.

CM training provides an environment to practice motivational interviewing techniques, a skill for eliciting behaviour change, by helping patients to explore and resolve ambivalence that is not addressed in most medical school curricula [21].

In addition to the improved counselling competencies, nutrition knowledge increased by 48% in the virtual cohort and by 41% in the in-person cohort. The CM elective vastly improved the most important requisites for successful care of patients with diet-related diseases.

A similar teaching success has already been described for in-person CM classes by Razavi et al. [20], Pang et al. [26], and Magallanes et al. [29]. A recent study by Razavi et al. [12] on virtual CM classes reported comparable outcomes.

The addition of CM to medical education is urgently needed as nutrition and dietetics and their translation to everyday life are underrepresented in medical schools. As published in a recent statement by the German Academy of Nutritional Medicine (DAEM), the German Society of Nutritional Medicine (DGEM), and the Association of German Physicians for Nutritional Medicine (BDEM), the skills and knowledge currently taught during medical school are not sufficient to provide adequate care based on the latest scientific findings [30]. This is underlined by the massive impact of over- but also malnutrition on healthcare systems.

The importance of nutrition counselling in medical practice was rated significantly higher after the CM elective in both cohorts. A qualitative study by Mogre et al. (2019) found that the average medical student underrated the need for physicians to learn about nutrition and to be concerned about the diet of their patients [31].

This underlines that the CM educational approach positively influences students’ perception and beliefs about the importance of this topic. Similar results were found by Razavi et al. [20]. Current literature suggests a perspective change towards nutrition in medical students. This will hopefully lead to future trainee doctors addressing their patients more frequently about nutrition in their professional life. Furthermore, CM classes with interprofessional staff may make medical students more strongly aware of the need for interprofessional cooperation across disciplines [11].

Students in both cohorts, who completed the CM elective, decreased the intake of unfavourable foods. This is consistent with results from Conroy et al. (2004) and Razavi et al. (2020), where students improved their eating habits after completing a CM class [20,32]. As physicians are role models for their patients, this aspect is also important for patient care.

In the study by Razavi et al. (2023), participants in the CM classes followed principles of the Mediterranean diet more strictly than a control group [20]. We were not able to prove this specific aspect in our cohorts. Another study found that attending a virtual series of nutrition lectures without hands-on cooking in a teaching kitchen improved students self-reported food choice [33].

No specific dietary recommendations were given to participants in this study. In the first module, the basics of healthy eating and nutrition recommendations for adults were discussed. Three diets were identified by the LEKuP [22] to address different health aspects best: a Mediterranean diet, the official nutrition recommendations of the German Nutrition Society, and a vegetarian diet. These diets can also be used for the dietary management of many diseases, so their principles were repeated frequently in the following modules.

Another limiting aspect is the fact that the virtual cohort took place during the COVID-19 pandemic. The pandemic changed eating habits in an unfavourable way [34] and increased food intake [35]. Regulations regarding on-campus teaching, personal contact restrictions, and restaurant visits were often adjusted and changed. This could possibly have also been an influencing factor on students eating behaviour.

The in-person cohort increased its intake of non-fried fish and legumes after completing the course. A possible hypothesis for this would be that these foods were also prepared in the teaching kitchen. In the virtual classes, only about two individually selected dishes were prepared at home by each student, while in the in-person class, between five to seven recipes were prepared together in the teaching kitchen. Since the students at home were allowed to choose the recipes themselves, it is possible that they primarily chose recipes that were close to their current eating habits (mere exposure effect).

In a study on eating behaviour and food choice, Wardle and Parmenter showed that nutrition knowledge was associated with healthy eating behaviour [36]. But motivation and practical skills are needed to translate knowledge into behaviour.

The students’ well-being improved significantly in both cohorts after the CM class (according to the WHO-5 well-being index). While the index has high clinimetric validity and its applicability across study fields is very high [37], external factors could still also have contributed to student well-being. This makes it difficult to distinguish whether the improvement in well-being was attributed directly to participation in the CM elective. In the virtual cohort, the lifting of the COVID-19 measures as well as seasonal changes may also have had an effect. Therefore, it is not possible to clearly determine the extent to which the CM classes improved student well-being.

The most unexpected result of the direct comparison between virtual and in-person CM classes was the on-par teaching success. In-person CM classes have more participant interaction, more recipes cooked, more social modelling, and an additional effect of the shared meals. It was assumed that this translates to a higher teaching success compared to the virtual format.

However, it must be considered that there are additional requirements for participants in the virtual format so that the total involvement is even higher compared to in-person (Table 1).

Students in the virtual cohort most likely look more precisely at the recipe cards and take more time to review the included dietary explanations, because they must select recipes themselves, shop for ingredients, and cook the dishes in their own kitchen. In addition, they must bear higher costs if they are not reimbursed for the additional grocery shopping. As the virtual CM classes were conducted during the COVID-19 pandemic, the hands-on teaching format was highly appreciated by the students because it was extremely interactive and used all senses compared to the usual digital broadcast of lectures (open feedback). There were no complaints about having to buy their own groceries, probably because of the included meal(s) cooked from the groceries (open feedback). Students could also cook in groups at someone’s home when allowed by pandemic regulations.

In the virtual cohort, registered dietitian nutritionists, the specially trained chefs, and tutors supervised the whole group at the same time. Therefore, questions, cooking instructions, and teaching of skills were given in parallel to all students. In the in-person cohort, the professional educators answered individual participants’ questions while everyone else kept cooking. It is possible that the information gain for individual participants was greater in the virtual cohort due to the accessibility of professional educators.

Taken together, this might compensate for the above-mentioned advantages of in-person CM cooking classes. The promising results of the virtual teaching kitchen format only apply to settings with participants who interactively cook hands-on in their own kitchens with video and audio turned on. Class sizes of more than 20 participants might be difficult to handle on one videoconference screen, and individual interaction diminishes with higher numbers of participants. An interactive hands-on cooking class delivered virtually should not be confused with large virtual webinars where hundreds of participants can take part at the same time. These formats almost completely lack interaction except the questions and answers stream and can better be compared to TV cooking shows.

When properly choreographed, virtual CM classes can have the same teaching effect as in-person classes. However, the total effort for participants is higher. This important result gives institutions much more flexibility in inventing CM. Medical schools can use the teaching format that best suits their local situation. When a larger teaching kitchen is lacking or driving distances in rural areas are long, the virtual teaching format can be used without limitations in effectiveness [12]. When medical students would have a choice between virtual and in-person CM classes, we expect that most students would choose in-person over virtual mainly because of the enhanced social experience, increased social support by peers and professional staff, and the better hands-on learning environment to improve cooking skills (open feedback).

Independent of the teaching format, CM classes counteract the inadequate and underprioritized nutrition education for medical students and enable health care professionals to properly address diet-related disease. Both in-person and virtual teaching kitchen platforms have promising effects when conducted with the same level of interaction. Especially virtual teaching kitchens cannot only reach students, but also patients, employees, retirees, or other members of the community in their homes or offices. The higher effort for virtual participants may in part or completely be compensated by less commuting time and less expenses for gas, electric power, or public transport depending on individual circumstances.

### Limitations of the Study

One limitation of the study is the relatively small sample size (virtual cohort n = 70; in-person cohort n = 80). However, most statistical results are already highly significant.

The teaching teams were similar in professional expertise and training at the three universities, but individual biases cannot be excluded.

Another limitation is that counselling competencies were self-reported and may contain a self-reported bias. Although students report a subjective improvement, it is difficult to assess how effectively they will translate these skills into clinical practice. Nevertheless, standardized comparative self-assessment questionnaires were used in all studies to evaluate teaching success of CM and similar interventions.

Inclusion of a control group that does not participate in CM classes could make the results even more robust. This should be implemented in further studies assessing the effectiveness of CM interventions.

Whereas it has clearly been shown that CM classes improve self-rated counselling competencies, this effect must ideally be verified. In a next step, it must be shown that better counselling on nutrition and lifestyle medicine and better interprofessional collaboration across disciplines as the ‘gold standard’ of comprehensive care produce better treatment outcomes [38,39].

## Figures and Tables

**Table 1:** Comparison of student time requirements between virtual and in-person.

	Virtual (Full Hours)	In-Person (Full Hours)
Planning session	1	1
7 × shopping for groceries	3.5	-
7 × preparing home kitchen	3.5	-
Setting up digital equipment in home kitchen (one time)	1	-
7 modules at 3 h each, virtual	21	-
7 modules at 3 h each, in-person	-	21
7 × cleaning home kitchen	3.5	-
7 × cleaning teaching kitchen	-	3.5
Mandatory case report	3.5	3.5
Total	37	29

**Table 2:** Baseline demographics of participants.

Participant Demographics	Virtual Cohort	In-Person Cohort	*p*-Value
Mean age (years ± SD)	24.3 (±6.7)	24.5 (±6.53)	0.55
Female/male/other (n)	57/13/0	57/23/0	0.147/0.147/-
Mean semester (±SD)	6.3 (±5.3)	6.26 (±4.26)	0.826
Completed professional training before starting medical studies (n)	29	31	0.998
Completed academic degree before starting medical studies (n)	5	8	0.556
City: Göttingen (n)	42 (60%)	22 (40.1%)	<0.001
City: Gießen (n)	12 (22.9%)	32 (27.5%)	0.002
City: Brandenburg (n)	16 (17.1%)	26 (32.5%)	0.085
N (total)	70	80	

**Table 3:** Mean changes in counselling competencies.

Topic	Before-Virtual M (SD)	After-Virtual M (SD)	Before-In-Person M (SD)	After-In-Person M (SD)	Virtual MD (SD)	In-Person MD (SD)
Mediterranean diet	3.41 (1.08)	4.23 (0.66)	3.18 (1.15)	4.3 (0.64)	0.81 (1.03) *	1.13 (1.04) *
Arterial hypertension diet	3.07 (1.21)	4.13 (0.74)	3.26 (1.09)	4.36 (0.57)	1.06 (1.24) *	1.1 (1.16) *
Vegetarian diet	3.60 (0.92)	4.41 (0.67)	3.54 (1.09)	4.34 (0.71)	0.81 (1.00) *	0.8 (0.96) *
Low-fat diet	3.37 (0.98)	4.13 (0.64)	3.43 (0.99)	4.19 (0.73)	0.76 (0.91) *	0.76 (1.02) *
High-protein diet	3.09 (1.02)	4.03 (0.72)	3.23 (1.06)	4.18 (0.67)	0.94 (1.08) *	0.95 (1.12) *
Serving size	3.06 (1.17)	4.00 (0.85)	2.9 (1.15)	3.95(0.72)	0.94 (1.30) *	1.05 (1.07) *
Moderate alcohol	3.71 (0.92)	4.23 (0.75)	3.70 (1.07)	4.44 (0.65)	0.51 (1.19) *	0.73 (1.00) *
Eating disorders	3.27 (1.12)	4.10 (0.80)	3.4 (1.05)	4.1 (0.73)	0.83 (1.17) *	0.7 (1.04) *
Cholesterol	3.30 (1.12)	4.01 (0.84)	3.19 (1.08)	3.94 (0.73)	0.71 (1.21) *	0.75 (1.02) *
Diabetes diet	3.22 (1.13)	4.27 (0.80)	3.04 (1.16)	4.21 (0.61)	1.04 (1.27) *	1.17 (1.14) *
Diabetes weight loss	3.14 (1.27)	4.37 (0.73)	3.29 (1.13)	4.36 (0.68)	1.23 (1.19) *	1.07 (1.45) *
Obesity weight loss	3.23 (1.13)	4.40 (0.67)	3.23 (1.18)	4.43 (0.63)	1.17 (1.22) *	1.20 (1.22) *
Omega fats ω-3 and -6	2.76 (1.26)	3.73 (0.82)	2.56 (1.15)	3.8 (0.84)	0.97 (1.31) *	1.23 (1.08) *
Dietary fats	2.84 (1.20)	3.87 (0.90)	2.65 (1.13)	3.83 (0.79)	1.03 (1.35) *	1.17 (1.22) *
Antioxidants	2.76 (1.13)	3.67 (0.94)	2.6 (1.15)	3.55 (0.89)	0.91 (1.14) *	0.95 (1.2) *
Calories	2.90 (1.23)	3.91 (0.81)	2.96 (1.14)	3.93 (0.89)	1.01 (1.21) *	0.96 (1.10) *
Hydration	3.6 (0.95)	4.27 (0.68)	3.7 (1.01)	4.43 (0.65)	0.67 (1.07) *	0.68 (1.10) *
Celiac disease	2.57 (1.23)	3.86 (0.86)	2.49 (1.28)	4.19 (0.79)	1.29 (1.33) *	1.7 (1.33) *
Food allergies	2.37 (1.12)	3.73 (0.78)	2.26 (1.12)	3.94 (0.78)	1.36 (1.25) *	1.67 (1.20) *
Glycaemic index	2.53 (1.28)	3.56 (0.97)	2.24 (1.16)	3.65 (0.87)	1.03 (1.41) *	1.42 (1.2) *
Fibre	3.11 (1.23)	4.20 (0.71)	2.9 (1.18)	4.29 (0.71)	1.09 (1.18) *	1.38 (1.18) *
Food label	2.87 (1.21)	4.04 (0.86)	2.79 (1.33)	4.16 (0.70)	1.17 (1.38) *	1.37 (1.25) *
Osteoporosis	2.40 (1.22)	3.86 (0.84)	2.44 (1.27)	3.89 (0.74)	1.46 (1.37) *	1.45 (1.32) *
BMI	3.61 (1.01)	4.37 (0.85)	3.60 (0.97)	4.55 (0.59)	0.76 (1.16) *	0.95 (0.98) *
Aerobic exercise	3.49 (1.14)	4.29 (0.84)	4.53 (1.1)	4.25 (0.75)	0.80 (1.25) *	0.72 (1.09) *
Overall	3.09 (1.13)	4.06 (0.78)	3.08 (1.17)	4.14 (0.72)	0.97 (1.20)	1.05 (1.13)

M = Mean, MD = Mean difference; (* *p* ≤ 0.001).

**Table 4:** Mean changes in attitudes towards nutrition counselling in medical practice.

Question	Before-Virtual M (SD)	After-Virtual M (SD)	Virtual MD (SD)	Before- In-Person M (SD)	After- In-Person M (SD)	In-Person MD (SD)
Nutrition counselling should be routine	4.36 (0.68)	4.56 (0.63)	0.2 (0.60) *	4.36 (0.815)	4.59 (0.650)	0.22 (0.94) *
Specific counselling can improve patients’ diet	4.76 (0.49)	4.86 (0.39)	0.1 (0.35) *	4.61 (0.72)	4.84 (0.37)	0.22 (0.71) *
Physicians’ counselling can improve patients’ diets	4.24 (0.79)	4.57 (0.65)	0.33 (0.76) *	4.38 (0.83)	4.74 (0.54)	0.36 (0.76) *

M = Mean, MD = Mean difference; (* *p* = 0.036 ≤ 0.001).

**Table 5:** Mean changes in nutrition knowledge.

	M of Right-Answering Participants before Virtual (%)	M of Right-Answering Participants after Virtual (%)	M of Right-Answering Participants before In-Person (%)	M of Right-Answering Participants after In-Person (%)	Virtual MD	In-Person MD
1. Recommended diet form	74.3	97.1	71.3	92.5	22.8 *	21.3 *
2. Carbohydrate percentage	31.4	84.3	38.8	61.3	52.9 *	22.5 *
3. Salt	34.3	81.4	25.0	61.3	47.1 *	36.3 *
4. Free sugars	62.9	70.0	56.3	76.3	7.1	20.0 *
5. Recommended protein	47.1	74.3	48.8	85.0	27.2 *	36.2 *
6. Malnutrition syndrome	58.6	81.4	65.0	85.0	22.8 *	20.0 *
7. Therapy obesity	81.4	91.4	68.8	86.3	10.0 *	17.5 *
8. Gout	62.9	77.1	67.5	66.3	14.2 *	-1.2
9. Monosaccharide gout	30.0	71.4	25.0	51.2	41.4 *	26.2 *
10. Dyslipoproteinemia	50.0	44.3	26.3	28.7	−5.7	2.4
11. Cereals for celiac disease	51.4	84.3	55.4	73.8	32.9 *	18.4 *
12. Chronic kidney disease therapy	58.6	68.6	56.3	61.3	10.0	5.0
13. Calcium oxalate stones	21.4	51.4	17.5	51.2	30.0 *	33.7 *
14. Fructose malabsorption	35.7	74.3	37.5	53.8	38.6 *	16.3 *
15. Omega-3 fatty acid	60.0	78.6	46.3	72.5	28.6 *	26.2 *
16. Calcium and Vitamin D	34.3	45.7	33.8	36.3	11.4	2.5
Overall	49.6	73.5	46.2	65.17	23.8	18.9

M = Mean, MD = Mean difference; (* *p* ≤ 0.034 ≤ 0.001; see Table S5 in Supplementary Materials).

**Table 6:** Mean changes in eating habits.

	Before-Virtual M (SD)	After-Virtual M (SD)	Virtual MD (SD)	Before-In-Person M (SD)	After-In-Person M (SD)	In-Person MD (SD)
Favourable foods	24.4 (4.07)	22.09 (3.83)	1.50 (3.09) *	20.83 (3.83)	21.52 (3.52)	−0.68 (3.37)
Unfavourable foods	10.72 (2.80)	10.0 (2.54)	0.72 (1.76) *	10.48 (2.79)	9.88 (2.21)	0.6 (1.76) *

M = Mean, MD = Mean difference; (* *p* = 0.012 ≤ 0.001).

## Data Availability

The data presented in this study are available on request from the corresponding authors. The data are not publicly available due to privacy restrictions.

## Supplementary Materials

The following supporting information can be downloaded at: https://www.mdpi.com/article/10.3390/nu15194281/s1, Table S1: Counselling competencies paired *t*-test; Table S2: Counselling competencies unpaired *t*-test; Table S3: Attitudes towards nutrition counselling in medical practice paired *t*-test; Table S4: Attitudes towards nutrition counselling in medical practice unpaired *t*-test; Table S5: Nutrition Knowledge paired *t*-test; Table S6: Nutrition Knowledge unpaired *t*-test; Table S7: WHO-5 well-being index (* *p* ≤ 0.001; ** *p* = 0.004); Table S8: WHO-5 well-being Index paired *t*-test; Table S9: WHO-5 well-being Index unpaired *t*-test; Table S10: Eating Habits Mean Difference; Table S11: Eating Habits paired *t*-test; Table S12: Eating Habits unpaired *t*-test.
