# Supplementary material for: Comparison of Effectiveness regarding a Culinary Medicine Elective for Medical Students in Germany Delivered Virtually versus In-Person
Source: Nutrients. 2023 Oct 8;15(19):4281. doi: 10.3390/nu15194281 (PMC10574049; doi:10.3390/nu15194281)
Supplement: Supplementary file 1 [file nutrients-15-04281-s001.zip › nutrients-2603744-supplementary.pdf]

Supplementary Materials

Table S1. Counselling competencies paired *t*-test.

Topic	T-Value (*t*), Virtual	Degrees of Freedom (df), Virtual	Significance (*p*) (2-Tailed), Virtual	Cohen's d, Virtual	T-Value (*t*), In-Person	Degrees of Freedom (df), In-Person	Significance (*p*) (2-Tailed), In-Person	Cohen's d, In-Person
Mediterranean Diet	−6.64	69	<0.001	1.03	−9.60	79	<0.001	1.04
Nutrition Arterial Hypertension	−7.14	69	<0.001	1.24	−8.44	79	<0.001	1.16
Vegetarian Diet	−6.83	69	<0.001	1.00	−7.45	79	<0.001	0.960
Low-Fat Diet	−6.97	69	<0.001	0.91	−6.67	79	<0.001	1.02
High-Protein Diet	−7.33	69	<0.001	1.08	−7.56	79	<0.001	1.12
Serving Size	−6.09	69	<0.001	1.30	−8.71	79	<0.001	1.07
Moderate Alcohol	−3.62	69	0.001	1.19	−6.57	79	<0.001	1.003
Eating Disorders	−5.94	69	<0.001	1.17	−5.97	79	<0.001	1.048
Cholesterol	−4.95	69	<0.001	1.21	−6.54	79	<0.001	1.025
Diabetes Diet	−6.88	69	<0.001	1.27	−9.18	79	<0.001	1.14
Diabetes Weight Loss	−8.61	69	<0.001	1.19	−8.39	79	<0.001	1.14
Obesity Weight Loss	−8.06	69	<0.001	1.22	−8.75	79	<0.001	1.14
Omega Fats ω-3 and -6	−6.21	69	<0.001	1.31	−10.23	79	<0.001	1.26
Dietary Fats	−6.37	69	<0.001	1.35	−8.61	79	<0.001	1.08
Antioxidants	−6.71	69	<0.001	1.14	−7.08	79	<0.001	1.22
Calories	−7.01	69	<0.001	1.21	−7.77	79	<0.001	1.2
Hydration	−5.23	69	<0.001	1.07	−5.54	79	<0.001	1.10
Celiac Disease	−8.08	69	<0.001	1.33	−11.39	79	<0.001	1.33
Food Allergies	−9.07	69	<0.001	1.25	−12.38	79	<0.001	1.20
Glycaemic Index	−6.08	69	<0.001	1.41	−10.53	79	<0.001	1.20
Fibre	−7.72	69	<0.001	1.18	−10.47	79	<0.001	1.18
Food Label	−7.08	69	<0.001	1.38	−9.78	79	<0.001	1.25
Osteoporosis	−8.90	69	<0.001	1.37	−9.82	79	<0.001	1.32
BMI	−5.46	69	<0.001	1.16	−8.67	79	<0.001	0.98
Aerobic Exercise	−5.37	69	<0.001	1.25	−5.94	79	<0.001	1.09

Table S2. Counselling competencies unpaired *t*-test.

	T-Value (*t*)	Degrees of Freedom (df)	Significance (*p*) (2-Tailed)	Cohen's d
Mediterranean Diet	1.83	148	0.69	1.03
Nutrition Arterial Hypertension	0.21	148	0.82	1.19
Vegetarian Diet	−0.89	148	0.92	0.97
Low-Fat Diet	0.43	148	0.66	0.95
High-Protein Diet	0.04	148	0.96	1.10
Serving Size	0.55	148	0.58	1.18
Moderate Alcohol	1.24	148	0.21	1.09
Eating Disorders	−0.71	148	0.47	1.10

Cholesterol	0.19	148	0.84	1.11
Diabetes Diet	0.67	148	0.50	1.20
Diabetes Weight Loss	−0.80	148	0.42	1.16
Obesity Weight Loss	0.14	148	0.88	1.22
Omega Fats (ω-3 and ω-6)	1.36	148	0.17	1.11
Dietary Fats	0.69	148	0.48	1.28
Antioxidants	0.18	148	0.85	1.17
Calories	−2.74	148	0.78	1.24
Hydration	0.09	148	0.92	1.09
Celiac Disease	1.89	148	0.60	1.33
Food Allergies	1.58	148	0.11	1.22
Glycaemic Index	1.85	148	0.66	1.30
Fibre	1.56	148	0.12	1.18
Food Label	1.78	148	0.07	1.23
Osteoporosis	−0.03	148	0.97	1.34
BMI	1.85	148	0.66	1.06
Aerobic Exercise	−0.39	148	0.69	1.16

Table S3. Attitudes towards nutrition counselling in medical practice paired *t*-test.

**Question**	**T-Value (*t*), Virtual**	**Degrees of Freedom (df), Virtual**	**Significance (*p*) (2-Tailed), Virtual**	**Cohen's d, Virtual**	**T-Value (*t*), In-Person**	**Degrees of Freedom (df), In-Person**	**Significance (*p*) (2-Tailed), In-Person**	**Cohen's d, In-Person**
Nutrition counselling should be routine	−2.77	69	0.007	0.60	−2.13	79	0.036	0.94
Specific counselling can improve patients' diet	−2.41	69	0.019	0.35	−2.83	79	0.006	0.71
Physicians' counselling can improve patients' diets	−3.64	69	0.001	0.76	−4.22	79	<0.001	0.76

Table S4. Attitudes towards nutrition counselling in medical practice unpaired *t*-test.

**Question**	**T-Value (*t*)**	**Degrees of Freedom (df)**	**Significance (*p*) (2-Tailed)**	**Cohen's d**
Nutrition counselling should be routine	0.191	148	0.849	0.801
Specific counselling can improve patients' diet	1.338	148	0.183	0.570
Physicians' counselling can improve patients' diets	0.272	148	0.786	0.761

Table S5. Nutrition Knowledge paired *t*-test.

**Question**	**T-Value (*t*), Virtual**	**Degrees of Freedom (df), Virtual**	**Significance (*p*) (2-Tailed), Virtual**	**Cohen's d, Virtual**	**T-Value (*t*), In-Person**	**Degrees of Freedom (df), In-Person**	**Significance (*p*) (2-Tailed), In-Person**	**Cohen's d, In-Person**

1. Recommended diet form	−4.19	69	<0.001	0.46	−3.83	79	<0.001	0.49
2. Carbohydrate percentage	−7.93	69	<0.001	0.56	−3.83	79	0.001	0.59
3. Salt	−6.77	69	<0.001	0.58	−5.59	79	<0.001	0.57
4. Free sugar	−0.87	69	0.388	0.69	−3.19	79	0.002	0.56
5. Recommended protein	−3.86	69	<0.001	0.59	−5.39	79	<0.001	0.6
6. Malnutrition syndrome	−3.93	69	<0.001	0.49	−4.13	79	<0.001	0.43
7. Therapy, obesity	−2.17	69	0.034	0.39	0.19	79	0.002	0.49
8. Gout	−2.30	69	0.024	0.52	−4.13	79	0.843	0.56
9. Monosaccharide gout	−5.76	69	<0.001	0.60	−3.14	79	<0.001	0.56
10. Dyslipoproteinemia	0.75	69	0.454	0.63	0.37	79	0.708	0.59
11. Cereals for celiac disease	−4.53	69	<0.001	0.61	−2.8	79	0.006	0.59
12. Chronic kidney disease therapy	−1.63	69	0.109	0.51	−0.78	79	0.436	0.57
13. Calcium oxalate stones	−4.20	69	<0.001	0.60	−6.01	79	<0.001	0.50
14. Fructose malabsorption	−5.64	69	<0.001	0.57	−2.7	79	0.008	0.53
15. Omega-3 fatty acid	−2.85	69	0.006	0.55	−4.13	79	<0.001	0.56
16. Calcium and Vitamin D	−1.43	69	0.159	0.67	−0.35	79	0.726	0.63

Table S6. Nutrition Knowledge unpaired *t*-test.

**Question**	**T-Value (*t*)**	**Degrees of Freedom (df)**	**Significance (*p*) (2-Tailed)**	**Cohen's d**
1. Recommended diet form	−0.206	148	0.837	0.477
2. Carbohydrate percentage	−3.211	148	0.002	0.577
3. Salt	−1.146	148	0.254	0.58
4. Free sugar	1.261	148	0.209	0.623
5. Recommended protein	0.936	148	0.351	0.594
6. Malnutrition syndrome	−0.381	148	0.704	0.458
7. Therapy obesity	1.02	148	0.309	0.449
8. Gout	−1.75	148	0.082	0.542
9. Monosaccharide gout	−1.588	148	0.114	0.583
10. Dyslipoproteinemia	0.818	148	0.415	0.613
11. Cereals for celiac disease	0.595	148	0.008	0.614
12. Chronic kidney disease therapy	0.315	148	0.139	0.601
13. Calcium oxalate stones	0.561	148	<0.001	0.567
14. Fructose malabsorption	0.622	148	0.012	0.585
15. Omega-3 fatty acid	0.433	148	<0.001	0.599
16. Calcium and Vitamin D	0.830	148	0.215	0.638

Table S7. WHO-5 well-being index (* $p \leq 0.001$; ** $p = 0.004$).

Mean Score before Virtual (SD)	Mean Score after Virtual (SD)	Mean Difference, Virtual (SD)	Mean Score before In-Person (SD)	Mean Score after In-Person (SD)	Mean Difference, In-Person (SD)
15.2 (3.60)	16.6 (3.37)	−1.4 (3.28) *	14.73 (4.89)	16.2 (4.49)	−1.46 (4.34) **

Table S8. WHO-5 well-being Index paired *t*-test.

T-Value, Virtual	Degree of Freedom, Virtual	Significance (*p*) (2-Tailed), Virtual	Cohen's d, Virtual	T-Value, In-Person	Degree of Freedom, In-Person	Significance (*p*) (2-Tailed), In-Person	Cohen's d, In-Person
−3.560	69	<0.001	3.289	−3.008	79	0.004	4.348

Table S9. WHO-5 well-being Index unpaired *t*-test.

T-Value	Degree of Freedom	Significance (*p*) (2-Tailed)	Cohen's d
0.098	148	0.922	3.890

Table S10. Eating Habits Mean Difference.

	Mean, Before Virtual	Mean, After Virtual	Mean Difference, Virtual	Mean, Before In-Person	Mean, After In-Person	Mean Difference, In-Person
Vegetables	4.37 (0.93)	4.33 (0.81)	0.04 (0.66)	4.13 (0.96)	4.30 (0.96)	−0.07 (0.85)
Legumes	2.40 (1.05)	2.56 (0.95)	−0.15 (0.75)	2.06 (0.81)	2.30 (0.64)	−0.23 (0.76) *
Fruit	3.99 (1.14)	4.00 (1.00)	−0.01 (0.78)	3.81 (1.08)	3.96 (1.08)	−0.15 (0.88) *
Nuts	2.87 (1.36)	3.03 (1.14)	−0.15 (1.07)	2.68 (1.05)	2.65 (1.04)	0.02 (0.92)
Milk products	3.56 (1.35)	3.47 (1.31)	0.086 (1.03)	3.75 (1.22)	3.61 (1.09)	0.13 (0.95)
Meat	1.80 (1.08)	1.60 (0.92)	0.20 (0.65) *	1.65 (0.90)	1.56 (0.77)	0.08 (0.48)
Fish	1.36 (0.56)	1.44 (0.62)	−0.08 (0.63)	1.24 (0.42)	1.44 (0.54)	−0.20 (0.48) *
Whole grain	3.36 (1.28)	3.51 (1.11)	−0.15 (1.16)	3.36 (1.07)	3.31 (1.08)	0.05 (1.2)
Vegetable oil	4.13 (0.90)	4.03 (0.90)	0.1 (0.93)	3.56 (1.017)	3.66 (1.03)	−0.10(1.01)
Alcohol	1.60 (0.62)	1.60 (0.66)	0 (0.45)	1.83 (0.725)	1.66 (0.655)	0.16 (0.43) *
Sweets	2.60 (0.95)	2.36 (0.94)	0.24 (0.71) *	2.64 (1.07)	2.54 (1.030)	0.10(0.75)
Calorie beverages	1.80 (1.04)	1.63 (0.87)	0.17 (1.00)	1.76 (0.79)	1.61 (0.738)	0.15 (0.57)
Butter	2.93 (1.34)	2.81 (1.20)	0.11 (1.02)	2.61 (1.21)	2.51 (1.07)	100 (0.88)
Favourable foods	24.4 (4.07)	22.09 (3.83)	1.50 (3.09) *	20.83 (3.83)	21.52 (3.52)	−0.68 (3.37)
Unfavourable foods	10.72 (2.80)	10.0 (2.54)	0.72 (1.76) *	10.48 (2.79)	9.88 (2.21)	0.6 (1.76) *

Differences in consumption frequency (* $p \leq 0.012$ compared to baseline).

Table S11. Eating Habits paired *t*-test.

	T-Value, Virtual	Degree of Freedom	Significance (*p*) (2-Tailed), Virtual	Cohen's d, Virtual	T-Value Präsenz	Degree of Freedom	Significance (*p*) (2-Tailed)	Cohen's d
Vegetables	0.536	69	0.594	0.669	−0.786	79	0.434	0.854
Legumes	−1.743	69	0.086	0.754	−2.769	79	0.007	0.767
Fruit	−0.151	69	0.880	0.789	−1.512	79	0.135	0.887
Nuts	−1.227	69	0.224	1.072	0.241	79	0.810	0.927
Milk	0.695	69	0.489	1.032	1.293	79	0.200	0.951
Meat	2.572	69	0.012	0.651	1.622	79	0.109	0.482

Fish	−1.136	69	0.260	0.631	−3.667	79	<0.001	0.488
Whole Grain	−1.131	69	0.262	1.163	0.373	79	0.710	1.2
Oil	0.895	69	0.374	0.935	−0.882	79	0.38	1.014
Alcohol	0	69	1.0	0.450	3.348	79	0.001	0.434
Sweets	2.858	69	0.006	0.711	1.182	79	0.241	0.756
Lemonade	1.425	69	0.159	1.007	2.330	79	0.22	0.576
Butter	0.929	69	0.356	1.029	1.016	79	0.313	0.880
Favourable	4.053	69	<0.001	3.096	−1.821	79	0.72	3.377
Unfavourable	3.446	69	<0.001	1.788	3.034	79	0.003	1.785

Table S12. Eating Habits unpaired *t*-test.

	T-Value, Virtual	Degree of Freedom	Significance (*p*) (2-Tailed)	Cohen's d
Vegetables	0.932	148	0.353	0.772
Legumes	0.645	148	0.520	0.765
Fruit	0.984	148	0.327	0.843
Nuts	−1.116	148	0.266	0.997
Milk	−0.32	148	0.750	0.989
Meat	1.212	148	0.227	0.567
Fish	1.249	148	0.214	0.559
Whole Grain	−1.070	148	0.286	1.182
Oil	1.250	148	0.213	0.977
Alcohol	−2.247	148	0.026	0.441
Sweets	1.187	148	0.237	0.735
Lemonade	0.162	148	0.871	0.805
Butter	0.092	148	0.927	0.952
Favourable, Before	−5.51	148	<0.001	3.950
Favourable, After	−2.286	148	0.024	3.674
Unfavourable, Before	−0.526	148	0.600	2.799
Unfavourable, After	−0.290	148	0.772	2.37

Liebe Studierenden,

vielen Dank für Ihre Bereitschaft an der Umfrage mitzuwirken!

Bitte generieren Sie als erstes einen persönlichen 10-stelligen Code gemäß nachfolgendem Muster:

Felder 1 und 2 sind für den Ort des Kurses. Felder 3 und 4 entsprechen jeweils dem zweiten Buchstaben Ihres Vornamens und Nachnamens, Felder 5 und 6 dem Tag ihres Geburtstages, Felder 7 und 8 den Initialen des Namens Ihrer Mutter. Die letzten beiden Felder 9 und 10 entsprechen den ersten beiden Buchstaben Ihres Geburtsortes.

Beispiel: Kurs in GÖttingen Frieda von Dannen, *17.12.1996; Wilma von Dannen, München

GÖ- R A 1 7 W D M Ü

Bitte generieren Sie Ihren Code hier *(Gilt nur für den Papierfragebogen):*

Teil A: Umfrageorganisation

Die folgenden Angaben dienen zur späteren Auswertung nach Kohorten

A1. Ihr Name: / Ihr Code:

Bitte generieren Sie als erstes einen persönlichen 10-stelligen Code gemäß nachfolgendem Muster:

Felder 1 und 2 sind für den Ort des Kurses. Felder 3 und 4 entsprechen jeweils dem zweiten Buchstaben Ihres Vornamens und Nachnamens, Felder 5 und 6 dem Tag ihres Geburtstages, Felder 7 und 8 den Initialen des Namens Ihrer Mutter. Die letzten beiden Felder 9 und 10 entsprechen den ersten beiden Buchstaben Ihres Geburtsortes.

*Beispiel:Kurs in GÖttingen Frieda von Dannen, *17.12.1996; Wilma von Dannen, München*

GÖ- R A 1 7 W D M Ü

A2. Kursnummer:

A3. Erhebungszeitpunkt der Umfrage

- Vorbefragung (vor Kursbeginn) ☐
- Nachbefragung (nach Kursende) ☐
- Sonderbefragung (ohne Zuordnung Kursbeginn/-ende) ☐

A4. Angabe des aktuellen Semester (Jahr)

- Sommersemester 2020 ☐
- Wintersemester 2020/2021 ☐
- Sommersemester 2021 ☐
- Wintersemester 2021/2022 ☐
- Sommersemester 2022 ☐
- Wintersemester 2022/2023 ☐

A5. In welchem klinischen Semester befinden Sie sich zur Zeit?

- 1 ☐
- 2 ☐
- 3 ☐
- 4 ☐
- 5 ☐

A6. Bitte geben Sie Ihr Geschlecht an.

- w ☐
- m ☐
- d ☐

A7. Wie alt sind Sie?

☐☐

A8. Haben Sie bereits eine Ausbildung vor dem Studium gemacht?Wenn ja, welche?

- JA, berufliche Ausbildung vor dem Medizinstudium ☐

Kommentar

- JA, akademische Ausbildung vor dem Medizinstudium ☐

Kommentar

NEIN, keine Ausbildung vor dem Medizinstudium ☐

Kommentar

☐

A9. Haben Sie bisher schon einmal an einem Kochkurs teilgenommen?

JA, an der Universität Göttingen ☐

JA, außerhalb der Uni Göttingen ☐

NEIN ☐

Teil B: Fragebogen zur ernährungsmedizinischen Beratungspraxis -Studierende-

B1. Ich koche selber

1x pro Monat/nie ☐

1-2 x pro Woche ☐

3-5 x pro Woche ☐

5-7 x pro Woche ☐

Täglich/ öfter ☐

B2. Ich esse (alle Gelegenheiten, zu Hause und außer Haus) ...

	1x pro Monat/nie	1-2 x pro Woche	3-5 x pro Woche	5-7 x pro Woche	Täglich/ öfter
Gemüse (z.B. Karotten, Spinat oder Tomaten)	☐	☐	☐	☐	☐
Hülsenfrüchte (z.B. Bohnen, Erbsen oder Linsen)	☐	☐	☐	☐	☐
Obst (z.B. Orangen, Äpfel oder Bananen)	☐	☐	☐	☐	☐
Nüsse oder Nussbutter (z.B. Erdnüsse, Mandeln, Walnüsse oder Cashews)	☐	☐	☐	☐	☐
Käse oder Milchprodukte (z.B. Joghurt)	☐	☐	☐	☐	☐
Rotes und verarbeitetes Fleisch (z.B. Wurst, Schinken, Kotelett, Döner, Hamburger, Steak, Hot Dogs usw.)	☐	☐	☐	☐	☐
Nicht-frittierter Fisch oder Meeresfrüchte (z.B. konserviert, gebacken oder gegrillt)	☐	☐	☐	☐	☐
Vollkornprodukte (z.B. Vollkornbrot, Vollkornnudeln, Hafer, brauner Reis)	☐	☐	☐	☐	☐
Pflanzenöle wie Rapsöl, Sonnenblumenöl und Olivenöl	☐	☐	☐	☐	☐
Eine Alkoholportion (entspricht 0,33l Bier/0,1 l Wein/2-4cl Schnaps)	☐	☐	☐	☐	☐
Süßes Gebäck (z.B. Kekse, Muffins, Kuchen, Doughnuts)	☐	☐	☐	☐	☐
Kalorienhaltige Getränke (z.B. Limonaden, Colagetränke, Fruchtsaftgetränke, Energydrinks)	☐	☐	☐	☐	☐
Butter, Kokosfett, Vollmilchprodukte	☐	☐	☐	☐	☐

B3. Meine persönliche Einstellung zur Ernährungsberatung in der ärztlichen Praxis ist...

	Stimme gar nicht zu	Stimme eher nicht zu	Teils/Teils	Stimmer eher zu	Stimme voll zu
Beratung zu Ernährungsfragen sollte Teil jeder ärztlichen Beratung sein, genau wie die Therapie und die Diagnose.	☐	☐	☐	☐	☐
Spezifische Empfehlungen zur Änderung des Essverhaltens können Patienten bei der Verbesserung ihrer Essgewohnheiten helfen.	☐	☐	☐	☐	☐
Ärzte können Einfluss auf die Essgewohnheiten ihrer Patienten nehmen, wenn sie sich Zeit nehmen, das Problem mit den Patienten zu besprechen.	☐	☐	☐	☐	☐

B4. Ich bin zuversichtlich, meine Patientinnen/Patienten zu folgenden Themen zu beraten...

	Stimme gar nicht zu	Stimme eher nicht zu	Teils/Teils	Stimmer eher zu	Stimme voll zu
Mediterrane Ernährung und ihr gesundheitlicher Effekt	☐	☐	☐	☐	☐
Die richtige Ernährung bei arteriellem Hypertonus	☐	☐	☐	☐	☐
Vegetarische Ernährung und ihr gesundheitlicher Effekt	☐	☐	☐	☐	☐
Fettarme Ernährung und ihr gesundheitlicher Effekt	☐	☐	☐	☐	☐
Eiweißreiche Ernährung und ihr gesundheitlicher Effekt	☐	☐	☐	☐	☐
Beratung zu richtigen Portionsgrößen	☐	☐	☐	☐	☐
Definition von moderatem Alkoholkonsum und dessen gesundheitlicher Effekt	☐	☐	☐	☐	☐
Das Erkennen von Warnzeichen und Symptomen von Patienten mit Essstörungen	☐	☐	☐	☐	☐
Die Rolle von (Nahrungs-)Cholesterin und gesättigten Fettsäuren auf die Blutlipide	☐	☐	☐	☐	☐
Ernährungsempfehlungen bei Diabetes Typ 2	☐	☐	☐	☐	☐
Bedeutung von angemessenem Gewichtsverlust für Diabetes Typ 2	☐	☐	☐	☐	☐
Strategien zur Gewichtsreduktion bei Adipositas	☐	☐	☐	☐	☐
Die Rolle von Omega-3 und-6-Fettsäuren in der Gesundheit des Herz-Kreislauf-Systems und jeweilige Ernährungsbeispiele	☐	☐	☐	☐	☐
Die Rolle verschiedener Nahrungsfette (gesättigte, etc.) und jeweilige Ernährungsbeispiele	☐	☐	☐	☐	☐
Das Erkennen von Antioxidantien-reichen Lebensmitteln	☐	☐	☐	☐	☐
Der spezifische Brennwert von Proteinen, Kohlenhydraten und Fetten und ihre Rolle in grundlegenden Stoffwechselprozessen	☐	☐	☐	☐	☐
Die Rolle von Flüssigkeitszufuhr in der Gesundheit und der Flüssigkeitsbedarf angepasst an Aktivität und Alter	☐	☐	☐	☐	☐
Zöliakie und Managementstrategien für Ernährung und Lebensalltag des Patienten	☐	☐	☐	☐	☐
Nahrungsmittel-Malabsorption und Managementstrategien für Ernährung und Lebensalltag des Patienten	☐	☐	☐	☐	☐
Die Rolle des glykämischen Index und der glykämischen Last in der diätetischen Behandlung	☐	☐	☐	☐	☐
Ballaststoffe in der Prävention von Krankheiten und Beispiele für entsprechende Lebensmittel	☐	☐	☐	☐	☐
Einschätzung von Gesamtkalorien, gesättigten Fettsäuren und Natriumgehalt mit Hilfe der Nährwertinformation bzw. Zutatenliste	☐	☐	☐	☐	☐
Osteoporose mit Prävention/Behandlungsstrategien für Ernährung und Lebensalltag des Patienten	☐	☐	☐	☐	☐
Die Bestimmung des BMI (body mass index) und der Waist-to-hip-ratio (geschlechtsspezifisch) bzw. des Bauchumfangs	☐	☐	☐	☐	☐
Der Gesamtnutzen (Effekt) von aerober körperlicher Aktivität auf Gesundheit und Wohlbefinden	☐	☐	☐	☐	☐

B5. **Welche Kostform wird im Leitfaden für Ernährungsmedizin in Klinik und Praxis (LEKuP) generell NICHT empfohlen?**

- Vegane Diät ☐
- Vegetarische Diät ☐
- Mittelmeer-Diät ☐
- Vollkost nach den DGE Empfehlungen ☐
- Konsistenzmodifizierte Vollkost ☐

B6. **Wie hoch sollte der Anteil von Kohlenhydraten an der Gesamtenergiezufuhr bei der vollwertigen Mischkost nach DGE sein (Empfehlung für Gesunde)?**

- 10 % ☐
- 20 % ☐
- 30 % ☐
- >50% ☐
- >60% ☐

B7. **Wie hoch ist die maximal empfohlene Gesamtmenge an Kochsalz pro Tag nach DGE (Empfehlung für Gesunde)?**

- 2,3 g ☐
- 3,5 g ☐
- 6,0 g ☐
- 10 g ☐
- 17 g ☐

B8. **Welcher Anteil an freien Zuckern (Prozent der Gesamtenergie) ist nach DGE akzeptabel?**

- Bis zu 5 EN% ☐
- Bis zu 10 EN% ☐
- Bis zu 15 EN% ☐
- Bis zu 20 EN% ☐
- Bis zu 40 EN% ☐

B9. **Auf wie viel g/kg Körpergewicht/Tag sollte die Proteinzufuhr bei einer schweren Mangelernährung angehoben werden?**

- bis zu 0,5 g ☐
- bis zu 0,8 g ☐
- bis zu 2,0 g ☐
- bis zu 3,4 g ☐
- bis zu 4,5 g ☐

B10. **Welches Syndrom kann bei der Therapie von schwer mangelernährten Personen auftreten?**

- Refeeding-Syndrom ☐
- Waisting-Syndrom ☐
- Metabolisches-Syndrom ☐
- Kurzdarm-Syndrom ☐
- Brugada-Syndrom ☐

B11. **Welche Maßnahme ist ausgehend von den Prinzipien der Vollkostformen zur Therapie von Adipositas <u>NICHT</u> angezeigt?**

- ein Energiedefizit von 500-600 kcal/Tag ☐
- reduzierte Fettzufuhr ☐
- Bevorzugung von Lebensmitteln mit hoher Energiedichte ☐
- wasserreiche Lebensmittel ☐
- Proteinzufuhr wie bei Vollkost oder ↑ ☐

B12. **Was ist bei der Ernährung bei Gicht/Hyperurikämie am wenigstens bedenklich?**

- Süßigkeiten ☐
- Alkoholfreies Bier ☐
- kohlensäurereiches Mineralwasser mit Zitrone ☐
- Limonaden ☐
- Innereien ☐

B13. **Welches Monosaccharid ist bei erhöhten Harnsäurespiegeln besonders problematisch?**

- Glucose ☐
- Fructose ☐
- Galactose ☐
- Alle Monosaccharide sind gleichermaßen problematisch ☐
- Monosaccharide haben keinen Einfluss auf die Harnsäurespiegel ☐

B14. **Welche diätetische Maßnahme ist bei einer Dyslipoproteinämie vom Typ der Hypertriglyceridämie <u>NICHT</u> indiziert?**

- Normalisierung des Körpergewichts ☐
- Reduktion der Aufnahme einfach ungesättigter Fettsäuren unter 10 % der Gesamtenergie ☐
- Erhöhung der Aufnahme mehrfach ungesättigter Fettsäuren über 10 % der Gesamtenergie ☐
- Begrenzung der Aufnahme von Mono- und Disacchariden ☐
- Erhöhung der Aufnahme von löslichen Ballaststoffen ☐

B15. **Welche Getreidesorte darf bei einer manifesten Zöliakie dennoch verzehrt werden?**

- [ ] Weizenvollkorn
- [ ] Gerste
- [ ] Dinkel
- [ ] Hirse
- [ ] Urgetreide (z.B. Urkorn, Einkorn und Emmer)

B16. **Welche Maßnahmen sollten bei chronischen Nierenerkrankungen stadienspezifisch beachtet werden?**

- [ ] Proteinrestriktion in frühen Stadien
- [ ] generelles Kaloriendefizit
- [ ] erhöhter Phosphatkonsum (zum Beispiel über einen gesteigerten Käsekonsum)
- [ ] Vitamin A-Substitution in allen Stadien
- [ ] kaliumreiche Ernährung in allen Stadien (z.B. durch vermehrten Konsum von Hülsenfrüchten)

B17. **Den Verzehr welcher Lebensmittel muss man bei Calciumoxalatsteinen NICHT vermeiden?**

- [ ] Rhabarber
- [ ] Schokolade
- [ ] Nüsse
- [ ] Spinat
- [ ] Eier

B18. **Was verbessert die Fruktoseverträglichkeit bei Patienten mit einer Fruktosemalabsorption?**

- [ ] Zugabe von Salz
- [ ] gleichzeitige Zufuhr von Glucose
- [ ] isolierte Aufnahme von fruktosehaltigen Lebensmitteln
- [ ] gleichzeitige Zufuhr von ungesättigten Fettsäuren
- [ ] gleichzeitige Zufuhr von Laktose

B19. **Welches dieser Öle enthält nur geringe Mengen Omega-3-Fettsäuren?**

- [ ] Rapsöl
- [ ] Walnussöl
- [ ] Leinöl
- [ ] Sonnenblumenöl
- [ ] Fischöl aus fettreichen Seefischen

B20. Welche Erkrankungen begründen keine Ausnahme für die empfohlene Kalziumzufuhr von 1000 mg/Tag und eine Vitamin D Zufuhr von 800 IE/Tag?

- Primärer Hyperparathyroidismus ☐
- glukokortikoidinduzierte Osteoporose ☐
- Kalziumoxalatsteine ☐
- Hyperkalzurie ☐
- aktive granulomatöse Erkrankungen ☐

Teil C: Fragen zum Wohlbefinden (WHO 5)

Zum Abschluss der Umfrage noch fünf letzte Fragen zu Ihrem Wohlbefinden

C1. In den letzten zwei Wochen...

	Die ganze Zeit	Meistens	Etwas mehr als die hälfte der Zeit	Etwas weniger als die Hälfte der Zeit	Ab und zu	Zu keinem Zeitpunkt
...war ich froh und guter Laune	☐	☐	☐	☐	☐	☐
...habe ich mich ruhig und entspannt gefühlt	☐	☐	☐	☐	☐	☐
...habe ich mich energisch und aktiv gefühlt	☐	☐	☐	☐	☐	☐
...habe ich mich beim Aufwachen frisch und ausgeruht gefühlt	☐	☐	☐	☐	☐	☐
...war mein Alltag voller Dinge, die mich interessieren	☐	☐	☐	☐	☐	☐

Vielen Dank für Ihre Teilnahme an der Umfrage! Wenn Sie uns noch ein kurzes Feedback in Schlagworten geben möchten, schreiben Sie uns bitte eine E-Mail an kursmanager@cookuos-ev.de Wir freuen uns auf Ihre Anregungen und Kritik.

Ihr Culinary Medicine Team
